# Reviewed article: Bezerra AB, Barrozo LV, Queiroz AP. Congenital heart diseases: population-based cross-sectional study and spatial analysis, São Paulo, 2012-2022. Epidemiol Serv Saude. 2026:35;e20250519

**DOI:** 10.1590/S2237-96222026v35e20250519.b

**Published:** 2026-03-16

**Authors:** Mariana Del Grossi Moura

**Affiliations:** 1 Universidade de Sorocaba, Sorocaba, SP, Brazil

## First round comments

Prezados autores,

Agradecemos a submissão do manuscrito RESS-2025-0519 intitulado “Cardiopatias congênitas no município de São Paulo: agrupamentos espaciais e estudo caso-controle dos fatores associados, 2012-2022” à Revista Epidemiologia e Serviços de Saúde: revista do SUS (RESS).

Após a revisão do seu artigo, identificamos a necessidade de uma revisão substancial (Major Revision) para que o manuscrito esteja de acordo com os padrões editoriais da revista e para viabilizar sua publicação.

O estudo apresenta limitações metodológicas relevantes, especialmente em relação ao delineamento, que comprometem a validade dos achados e das interpretações:

1. Delineamento inadequado para o objetivo proposto: embora o estudo se declare como caso-controle de base populacional, os controles foram selecionados sem garantia de que não apresentavam cardiopatia congênita, uma vez que não houve triagem diagnóstica específica na população geral. Sugiro uma reflexão quanto à caracterização metodológica do estudo: considerando o uso de dados secundários de base populacional, a ausência de seguimento temporal e a inexistência de confirmação diagnóstica sistemática para os controles, o delineamento de estudo transversal com análise espacial integrada, seria mais adequado.

2. Recomenda-se que a seção de métodos seja revista e estruturada de acordo com o checklist STROBE (Strengthening the Reporting of Observational Studies in Epidemiology), que orienta a adequada descrição de estudos observacionais. A adoção desse guia permitirá aprimorar a apresentação do delineamento, critérios de seleção, definição de variáveis, fontes de dados, estratégias para controle de viés, tamanho amostral e métodos estatísticos, garantindo maior transparência e rigor metodológico. O checklist está disponível em: https://www.strobe-statement.org/.

3. Esclarecer se foi considerado algum procedimento metodológico para controle ou ajuste de potenciais fatores de confusão contextuais, especialmente diante da integração de uma variável territorial agregada (residência em cluster) em modelos de regressão logística em nível individual.

4. A seção “Aspectos éticos” deve ser retirada do texto completo e apresentada em formato de quadro na

folha de rosto, conforme as normas editoriais da RESS. Consulte as instruções aos autores disponíveis no site da RESS.

5. Esclarecer de forma objetiva se a variável utilizada na análise espacial e na construção da variável “área de residência” corresponde ao local de residência da mãe ou ao local de ocorrência do parto, conforme registrado no SINASC. Essa distinção é fundamental, uma vez que a interpretação das associações territoriais e a confiabilidade da análise espacial dependem diretamente da correta identificação do território de residência. Caso tenha sido utilizado o local de ocorrência, recomenda-se revisar a interpretação dos achados e incluir essa limitação explicitamente na discussão do manuscrito

6. Em alguns momentos, o texto afirma associações sem ponderar adequadamente a magnitude modesta ou a proximidade dos limites de significância - por exemplo, o número de consultas de pré-natal (OR 1,05; IC95% 1,00-1,10). Recomenda-se qualificar essas interpretações para não superestimar a importância prática dos achados.

7. É imprescindível que o manuscrito explicite essas limitações na Discussão, especialmente quanto: à possível subnotificação de casos entre os controles; ao viés decorrente do uso de variável territorial agregada; à ausência de controle de fatores contextuais.

8. Embora o texto reconheça algumas limitações, seria importante reforçar que, por tratar-se de um estudo transversal com base em registros secundários, não é possível inferir relações causais, apenas associações.

9. Recomenda-se que as figuras e tabelas sejam acompanhadas de títulos completos e autoexplicativos, que descrevam o conteúdo apresentado, os métodos aplicados (ex.: técnica de varredura espacial, modelo de Poisson), o número de clusters identificados e o período analisado. Além disso, sugere-se incluir nas legendas ou no próprio mapa a identificação dos distritos por nome ou descrição correspondente aos números apresentados, para facilitar a interpretação sem necessidade de recorrer ao texto. A clareza das ilustrações é essencial para que o leitor compreenda os achados.

Solicitamos que todos os comentários da revisão por pares sejam respondidos pontualmente por meio de uma carta resposta detalhada, explicando as modificações realizadas ou justificando, quando necessário, a manutenção do texto original. A nova versão do manuscrito será reconsiderada após essa reformulação

e poderá passar por nova rodada de avaliação

Recommendation: Major revision

## Second round comments

Prezados autores,

Agradecemos a submissão do manuscrito RESS-2025-0519.R1 intitulado “Cardiopatias congênitas: agrupamentos espaciais e fatores maternos e obstétricos associados, município de São Paulo, 2012-2022” à Revista Epidemiologia e Serviços de Saúde: revista do SUS (RESS).

Parabenizamos pelo avanço na revisão do manuscrito. Após a análise da nova versão, verificamos que a maior parte das recomendações foi plenamente atendida. No entanto, identificamos alguns ajustes finais necessários:

- Título: deve informar o tema principal, delineamento, local e ano(s) da pesquisa, em consonância com o guia de redação aplicável. Títulos devem ser diretos, objetivos e sem siglas. A pontuação aceita no título são dois pontos (:), para indicar após o tema principal o delineamento, local e ano(s); Sugestão “Cardiopatias congênitas: estudo transversal de base populacional e análise espacial, município de São Paulo, 2012-2022” - Introdução: ainda há referência ao “delineamento caso-controle”. Como o estudo foi reclassificado para “transversal de base populacional com análise espacial”, recomendamos ajustar esse trecho para garantir coerência metodológica.

- Objetivo do estudo: há uma divergência entre o resumo e a introdução, onde são usados termos diferentes (“avaliar” vs “analisar”; “características maternas e obstétricas” vs “fatores sociodemográficos e obstétricos”).

Sugerimos padronizar a redação, utilizando a mesma formulação em ambos os locais.

- Terminologia: como foi mantido o uso de “raça/cor” e “agrupamento”, em lugar de “raça/cor da pele” e “aglomerado”, sugerimos que essa decisão seja explicitada na seção de Métodos, de forma breve, para que o leitor compreenda a escolha terminológica em consonância com o SINASC/IBGE. Por exemplo: “A variável raça/cor foi utilizada conforme registro padronizado do SINASC (branca, preta, parda, amarela, indígena), mantida a nomenclatura oficial do sistema. Para a análise espacial, adotou-se o termo ‘agrupamento’... “Solicitamos que todos os comentários da revisão por pares sejam respondidos pontualmente por meio de uma carta resposta detalhada, explicando as modificações realizadas ou justificando, quando necessário, a manutenção do texto original.

Recommendation: Minor revision

